# Is There Any Relation between Serum Levels of Interleukin-10 and Neurophysiological Abnormalities in Bell’s Palsy?

**DOI:** 10.15388/Amed.2021.28.2.15

**Published:** 2021-08-31

**Authors:** Mehdi Maghbooli, Abdolreza Esmaeilzadeh, Fatemeh Karami Zarandi, Arezoo Jafarzadeh, Sajjad Biglari, Nazanin Azizi Shalbaf, Negar Farhoudi

**Affiliations:** Zanjan University of Medical Sciences, Vali-e-Asr University Hospital, Neurology Department, Zanjan, Iran ORCID ID: https://orcid.org/0000-0003-0482-9062; Zanjan University of Medical Sciences, Immunology Department and Cancer Gene Therapy Research Center, Zanjan, Iran ORCID ID: http://orcid.org/0000-0002-5402-3967; Zanjan University of Medical Sciences, Vali-e-Asr University Hospital, Neurology Department, Zanjan, Iran; Zanjan University of Medical Sciences, Zanjan, Iran; Zanjan University of Medical Sciences, Zanjan, Iran; Zanjan University of Medical Sciences, Zanjan, Iran ORCID ID: http://orcid.org/0000-0001-8872-0093; Zanjan University of Medical Sciences, Vali-e-Asr University Hospital, Neurology Department, Zanjan, Iran

**Keywords:** Bell’s palsy, Interleukin-10, Nerve conduction study

## Abstract

**Background:**

Bell’s palsy is the most common cause of peripheral facial palsy. The etiology and treatment of Bell’s palsy are still controversial. Previous studies emphasize the role of herpes simplex and herpes zoster viruses in this ailment. The role of Interleukin-10 (IL-10) in Bell’s palsy is yet unknown, and few studies have shed light on the matter. This study intended to assess the prognostic value of IL-10 and its relation to the intensity of electrodiagnostic abnormalities and evaluate its potential use as a factor for judging the need for medical or surgical interventions.

**Materials and Methods:**

30 patients in the acute phase of Bell’s palsy participated in this study. Peripheral blood samples were obtained for IL-10 assessment within the first 72 hours (before commencing treatment), and a nerve conduction study (NCS) was performed six days after symptom onset.

**Results:**

There was no significant correlation between IL-10 serum levels and the severity of nerve conduction pathology in Orbicularis oculi and Orbicularis oris muscles. Also, IL-10 serum levels did not show any meaningful relationships with participants’ age, gender, or symptoms.

**Conclusion:**

The IL-10 serum levels are not relevant to the pathology of Bell’s palsy, and the assessment of IL-10 serum levels cannot be used as an alternative to NCS for evaluating the severity of acute Bell’s palsy.

## Abstract (lt)

**Ar interleukino 10 koncentracija serume sukelia neurofiziologinių sutrikimų sergant Belo paralyžiumi:**

**Santrauka. *****Problema.*** Belo paralyžius dažniausiai sukelia periferinį veido paralyžių. Belo paralyžiaus etiologija ir gydymas vis dar yra kontroversiški. Ankstesniuose tyrimuose pabrėžiama pūslelinės ir juostinės pūslelinės svarba sergant šia liga. Interleukino 10 (IL-10) vaidmuo sergant Belo paralyžiumi iki šiol nežinomas – atlikta tik keletas tyrimų. Šio tyrimo tikslas – įvertinti prognozinę IL-10 vertę ir tai, ar jis gali būti siejamas su elektrodiagnostinių sutrikimų intensyvumu, bei galimybę naudoti jį kaip veiksnį sprendžiant dėl medicininio ar chirurginio gydymo.

***Medžiaga ir metodai.*** Tyrime dalyvavo 30 pacientų, sergančių ūminiu Belo paralyžiumi. Periferinio kraujo mėginiai IL-10 tyrimui buvo paimti per pirmąsias 72 valandas (prieš pradedant gydymą), o nervų laidumo tyrimas (angl. *NCS*) atliktas praėjus šešioms dienoms nuo simptomų atsiradimo.

***Rezultatai. ***Aiškios koreliacijos tarp IL-10 koncentracijos serume ir žiedinio akies raumens ir žiedinio burnos raumens nervų laidumo patologijos sunkumo nenustatyta. Be to, IL-10 koncentracija serume neatskleidė jokio reikšmingo dalyvių amžiaus, lyties ar simptomų ryšio.

***Išvada.*** IL-10 koncentracija serume nėra reikšminga Belo paralyžiaus patologijai, o IL-10 koncentracijos serume tyrimas negali būti laikomas NCS, vertinant ūminio Belo paralyžiaus sunkumą, alternatyva.

**Raktažodžiai: **Belo paralyžius, interleukinas 10, nervų laidumo tyrimas

## Introduction

Bell’s palsy is the most common cause of peripheral facial palsy [1]. The annual incidence of Bell’s palsy is 14-25 cases per hundred thousand individuals in a population [2]. Its incidence increases somewhat with age, and there is a slight difference between genders [3]. Also, its incidence partially increases in the winter [4]. Pathologically, all patients have some degree of nerve fiber degeneration; however, the underlying etiology is not unanimously agreed upon; for example, suspicions of viral agents being a supposed trigger have been proposed many times, but the nature of this mechanism has only been established in recent years. The Herpes simplex virus genome has been detected in the geniculate ganglia of Bell’s palsy patients. However, controversy exists regarding the immediate cause of the paralysis, whether it is only a result of viral infection or an ischemic neuropathy secondary to infection [5].

The etiology and treatment of Bell’s palsy are still controversial. Some of the proposed causes are inflammation, autoimmunity, viral infections, and ischemia [6].

Serological studies indicate that a high percentage of people with Bell’s palsy possess herpes simplex antibodies (compared to controls) [7]. However, because there is no clear evidence on increasing of the number of specific antibodies in Bell’s palsy, the disease can result from the delayed activation of the virus. This theory is supported by increased serum interferon during Bell’s palsy [8,9].

Some studies suggest that Bell’s palsy may have an autoimmune basis. Recent evidence shows that the amount of serum complements increases. In addition, during the first 24 hours, the number of peripheral blood T lymphocytes reduces, and B lymphocytes increase. Nevertheless, there were no differences in the total number of lymphocytes reported, and a relation between these increases and patient recovery time was not established [10]. Thus, Bell’s palsy may be an autoimmune demyelinating cranial neuritis. In most cases, it is a mononeuropathy variant of Guillain–Barré syndrome [11].

Determining facial nerve function in Bell’s palsy using electrical tests like Electroneuronography (ENoG) is accompanied by various errors [12]. Assessment difficulty in bilateral cases, problematic electrical stimulation of two points due to the high degree of nerve anastomosis in the parotid gland, a short period between excitations, and the complexity of the arrangement of facial muscles are some examples. It seems that measuring the compound muscle action potential (CMAP) amplitude is a reasonable way to assess the seventh pair of cranial nerves in Bell’s palsy [13,14].

Cell-mediated immune response in Bell’s palsy has been conveyed [15, 16]. Also, there is an evidence of changes in levels of some subtypes of lymphocytes in its acute phase [8]. Furthermore, reductions in T (CD3) and T Helper (CD4) cells in patients with Bell’s palsy compared to healthy subjects have been established. This indicates the role of immune response in Bell’s palsy [17], as much as somebody regards it as autoimmune neuritis [18].

Evaluation of patients with Bell’s palsy has shown the increase in serum levels of IL1, IL6, and TNF-alpha (Tumor Necrozing Factor) compared to healthy individuals, representing the activities of the cell-mediated immune system’s operative factors [19]. Therefore, this theory suggests that the disease occurs due to the humoral immune response to a viral infection. In addition, one study on 25 children afflicted by Bell’s palsy revealed a high Neutrophil to Lymphocyte Ratio (NLR) and supported the inflammatory nature of this disease [20].

Interleukin-10 (IL-10) is an anti-inflammatory cytokine that plays an essential role in immune suppression and preventing various autoimmune diseases. IL-10 plays two significant roles: inhibiting cytokine production from macrophages and macrophage inhibition during T lymphocytes activation [21]. IL-10 secretion increases during infections, leading to clearance of pathogens and healing of damaged tissue. Also, there were reports about increased levels of serum IL-10 during autoimmune diseases [22].

The role of IL-10 in Bell’s palsy is unknown, and there have not been any studies done hereupon. Based on recommendations for treating patients with only severe types of the disease and the increase of IL-10 levels in the acute phase of viral diseases and their role as the leading cause of this affliction, this study was designed to assess the relationship of IL-10 with the intensity of electrodiagnostic abnormalities (as an established prognostic factor) in Bell’s palsy and to evaluate its possible use as a factor for assessing the need for medical or surgical interventions.

## Materials and Methods

After receiving ethical approval from Zanjan University of Medical Sciences (IR.Zums.Rec.1395.49), this cross-sectional study was carried out after obtaining written consent from participating patients.

The study was conducted in Vali-e-Asr Hospital in Zanjan, Iran. General and demographic information was gathered via a questionnaire. Blood samples (5 ml) were obtained from patients within the first 72 hours after diagnosis, just before starting treatment. These samples were centrifuged, and serum was stored at 80 ^o^ C to measure Interleukin-10 levels. Nerve conduction studies (NCS) were implemented on the 6th day following Bell’s palsy onset. We used the Boster Immunoleader HTL10 ELISA kit to measure serum IL-10 levels. The normal range was <3.0 pg/ml, according to the kit manual.

Thirty patients afflicted by acute Bell’s palsy were included after being referred to the Neurology clinic and emergency department of Vali-e-Asr Hospital in Zanjan. The sampling method was a consecutive and simple nonrandomized selection. Inclusion criteria include proven idiopathic paralysis along with the exclusion of other causes of facial nerve palsy such as direct trauma, otitis media, brain stem, and malignant lesions. Patients with systemic infections were also excluded. All possible etiologies were ruled out via accurate history acquisition and systemic and neurologic examinations. 

The proportion of the affected side’s CMAP amplitude to that of the healthy side was assessed via supramaximal stimulation of Orbicularis Oris or Orbicularis Oculi muscles. The scale of the assessment was the CMAP amplitude. Lower calculated amplitude ratios were indicators of lower intensity involvement.

This study encountered limitations such as lack of accessible patients in a suitable time interval, the lack of Electromyography (EMG) and Blink Reflex exams, and uncertain accuracy in timing for subsequent examinations after onset. Also, we could not follow patients after 3 to 6 months from the initiation of the study.

All data were analyzed through frequency tables, Index of dispersion, Central Tendency, Odds Ratios, and a Confidence Interval (CI) of 95% was calculated (SPSS 18 .0 software). A P-value lower than 0.05 was considered to be statistically significant. In addition, linear regression, One Way ANOVA, and Chi-square tests were used to analyze results.

## Results

### Descriptive data

Among 30 patients participating in this study, 19 were men and 11 women with a mean age of 45±17 years (the youngest patient was 20 years old and the oldest one 80 years old). Given the history of atherosclerotic diseases, nine patients (30%) had a positive documented history of diabetes mellitus, hypertension, hyperlipidemia, stroke, and ischemic heart diseases. Only one patient (3.3%) had a positive family history. Facial nerve palsy occurred on the right side in 13 patients (43.3%) and on the left side in 17 patients (56.7%). The frequency distribution of symptoms accompanied by a disease such as diarrhea or cold one week before onset is shown in Table 1. 

**Table 1.** Frequency distribution of the subjective and objective clinical findings Diarrhea/common cold

Symptoms/Signs	Number of Participants	Percentage of Total Participants
Diarrhea/common cold	1	3.3
Postauricular pain	18	60
Taste disturbance	11	36.7
Hyperacusis	5	16.7
Hyperlacrimation	18	60

### Outcome data

All participants underwent NCS of Orbicularis Oris and Orbicularis Oculi muscles on day 6 of the disease onset, calculated based on the amplitude of the compound muscle action potential (CMAP) on the affected compared to the healthy side. Then they were divided into three groups: good prognosis (amplitude ratio >50%), moderate prognosis (ratio of 10 to 50%), and poor prognosis (ratio <10%). This ratio is calculated for both muscles and is based on the maximum intensity of severity. Serum levels of Interleukin-10 within the first 72 hours from disease onset were measured via ELISA. The lowest reported level of IL-10 was 0.0, and the highest level was 6.8, with a mean level of 1.97 ± 1.82 pg/ml. P-value less than 0.05 was regarded as statistically significant, and IBM SPSS 18.0 software for Windows was used for statistical analysis.

### Main results

According to the aforementioned CMAPs ratio in the O. oculi muscle, the minimum ratio was 0.03, and the maximum was 0.92. In the O. oris muscle, the lowest ratio was 0.04 (most severe), and the highest ratio was 0.97 (least severe). Based on the NCS results for both muscles, poor, moderate, and good prognostic groups comprised 9, 9, and 12 patients, respectively. On the other hand, classification based on the most severe injury in each of the O. oris and O. oculi muscles disclosed that 15 patients would have a poor prognosis, 7 a moderate one, and 8 a good one. 

At a glance, the relationship between severity and the gender of patients is as follows: the poor prognosis group included four males and five females, the moderate prognosis group had seven males and two females, and the good prognosis group consisted of 8 men and four women. The chi-square test results indicated no significant relationship between patients gender and disease severity in the O. oculi muscle (p=0.325). Evaluation of this relationship in the O. oris muscle and the relation between the highest involvement rate (based on NCS) and gender showed no significant relationship. In a complete survey, we could not demonstrate any meaningful interrelation between age, affected side, family history, past medical history, postauricular pain, flu-like symptoms, diarrhea, taste disorders, hyperacusis, and hyperlacrimation with the intensity of injury in O.oculi and O.oris muscles. 

The linear regression test indicated no meaningful relationship between age and serum levels of IL-10 (P-value=0.326). The mean serum level of IL-10 in men was 2.06 ±1.88 pg/ml with a minimum level of 0.0 and a maximum of 6.8. In women, the mean serum level of IL-10 was 1.80±1.78 pg/ml with the lowest and highest levels of 0.0 and 5.8, respectively. ONE-WAY ANOVA revealed no significant relationship between serum levels of IL-10 and the gender of patients (P-value=0.704). Also, none of the historical and symptomatic factors indicated any significant correlation with serum levels of IL-10.

We studied the relationship between the IL-10 serum levels and the NCS results in both O. oculi and O. oris muscles in all participants. We found no relationship between them in the linear regression test (P =0.341 and 0.523, respectively). Furthermore, analysis by linear regression did not declare any relationship between the lowest CMAP amplitude ratio (severe damage) in each of the O. oris and O. oculi muscles and serum levels of IL-10 (P-value=0.472). 

Table 2 represents the main findings regarding the serum levels of IL-10 in poor, moderate, and good prognosis groups based on the NCS results of O. oculi and O. oris muscles. Analysis via ONE-WAY ANOVA did not demonstrate a significant association.

**Table 2.** Comparison of Serum Levels of IL-10 between NCS prognostic groups in each muscle

NCS‡	Prognosis group	Serum level of IL-10†			P-value (one-way ANOVA)
		mean	Minimum	Maximum	
Orbicularis oculi muscle	poor	2.43±1.72	0.0	6.40	
	moderate	1.31±1.42	0.0	3.76	2.46
	good	2.47±1.86	0.0	4.81	
Orbicularis oris muscle	poor	1.89±2.28	0.0	6.80	
	moderate	1.24±1.31	0.0	4.00	2.62
	good	2.57±1.79	0.0	5.80	
Maximum intensity of involvement	Poor	1.47±1.46	0.0	3.80	
	moderate	2.36±2.04	0.3	6.80	0.594
	good	2.04±1.96	0.0	5.80	

†: interleukin-10

‡: nerve conduction study

## Discussion

This investigation was a cross-sectional study aimed at determining Interleukin-10 serum levels in the acute phase of Bell’s palsy and evaluating its interrelation with the severity of abnormalities settled in the nerve conduction study. In this study, the least amplitudes were selected among the results of NCS for O. Oculi, and O. Oris muscles, and the relevance of “afflicted: normal” CMAP amplitude ratio to the serum levels of interleukin-10 was investigated. This survey did not lead to any signifi-cant association. Due to the lack of significance of this relation in O. Oculi and O. Oris muscles, it can be deduced that serum level of IL-10 is not interrelated to the severity of nerve damage, even with changing a tested muscle.

Fitzgerald et al. used a cytometric bead array to examine the expression of various cytokines produced by splenocytes stimulated with antibody to CD3 (anti-CD3) and anti-CD28 in the presence or absence of exogenous IL-27 during in vitro studies. It showed that IL-27 induced production of IL-10 by effector T cells and concluded that the suppression of autoimmune inflammation of the central nervous system occurs due to IL-10 secreted by IL-27stimulated T cells [23]. 

Mark Mey et al. studied the relation between Evoked Electromyography (EEMG) and idiopathic facial nerve palsy in 1983. They compared the amplitude of responses to EEMG stimulus between one side of the face and another in 288 healthy individuals and found less than 50% difference in all participants. Therefore, they concluded that the normal range of amplitude differences between the two sides should be less than 50%. Then they conducted the same evaluation in 50 Bell’s palsy patients referred within the first 14 days of onset. As a result, they included those patients whose affected/healthy side amplitude ratio was more than 25% in a good prognosis group. Their study reported a complete recovery for 36 patients (92%) in the aforementioned group [24].

In a study by Jori et al. which was about the prognostic value of nerve conduction velocity (NCV) in Bell’s palsy in Poland in 1998, NCV was measured in 30 healthy individuals (both sides of the face) and in 51 patients with unilateral Bell’s palsy, with normal range contemplated 47.8±5.1m/s. Results revealed that most of the subjects with NCV less than 30 milliseconds did not reach complete recovery. However, in participants with normal NCV, the degeneration degree was less than 60%, demonstrated by electroneurography [25].

A survey about IL-10 gene polymorphism and Sasang structure in Bell’s palsy performed by Jong et al. in 2005 in Korea showed herpes virus infection as the most common cause of Bell’s palsy with an increased level of IL-6, IL-8, and TNFα. They compared 62 patients afflicted by Bell’s palsy with 104 healthy individuals (control group). There was no statistical difference in IL-10 gene polymorphism between control and case groups [26].

In 2013 Gerd Fabian Volk et al. evaluated time forecasting and remission of 259 patients with acute facial palsy in Germany. They recommended that facial nerve electromyography is a powerful modality for time forecasting of palsy remission. The study’s findings showed that 24% of patients had normal EMG and increased latency and decreased amplitude in 76% of them. Also, patients with spontaneous pathologic activity and a severe decrease in voluntary activity on EMG had poorer outcomes and decreased recovery rates [27].

Parkash KM et al. found a strong relationship between the degree of facial nerve degeneration (evaluated by NCS) and the short-time prognosis of Bell’s palsy (the first month of the disease). They evaluated facial NCV in the first and second months of the disease and disclosed that NCV in the second month is not as efficient as in the first month [28].

Although our study settled that the appropriate time for evaluating IL-10 levels is 72 hours after disease initiation, determining the significance of the increase in serum levels of IL-10 was not possible because we did not have a control group.

In previous studies, NCS was used to assess the severity of Bell’s palsy or as a prognostic factor of the disease. Ceccanti and colleagues studied the predictive role of neurophysiology in Bell’s palsy in 2013. Ninety-two patients were enrolled, and NCS was performed for Orbicularis Oris and Orbicularis Oculi muscles. They concluded that amplitude reduction in the affected side compared to the healthy side was related to the outcome of Bell’s palsy [29]. Joachims and colleagues’ study performed on 100 patients with Bell’s palsy found that NCS has a significant role in early diagnosis and determining the severity of the disease. More disturbances in NCS indicated minor improvement [30]. In our study, NCS results were used to assess the severity and prognosis of patients and categorize them. However, this parameter did not turn out to be the basis for the designation of response to treatment and remission. It was also not re-evaluated, which implies the limitation of our study compared with the others.

In the present study, the intensity of nerve palsy was assessed according to NCS results. The variables include age, gender, affected side, family history, history of underlying disease, symptoms, abnormal taste, hyperacusis, and tearing. There was no relationship between these variables and NCS findings. In order to determine the prognostic value of electrophysiology of Bell’s palsy in O. Oculi muscle and Nasolabial fold, Sang et al. reviewed the electroneurographic results of 81 patients at the beginning and end of the study. They did not see any significant association between electroneurographic results and age, sex, affected side, and time interval from ENoG [31]. Thus, this study is concordant with our findings on the lack of relation between NCS results and said variables.

Takashi and colleagues conducted a study in 2014 on the prognostic factors of Bell’s palsy with 679 patients. Effects of age, gender, affected side, past medical history, and severity were assessed on disease outcome. Disease severity was relevant to nonhealing conditions in one week. Age, sex, affected side and underlying diseases had no relevance with prognosis [32]. The results of our study also showed a lack of association between the intensity of nerve injury with said variables. 

In another study, Mehvari and colleagues attempted to highlight the role of electrodiagnosis and taste disturbance in the prognosis and diagnosis of Bell’s palsy. Their study recruited 44 patients with Bell’s palsy, and the primary factor was amplitude reduction in the affected side. Patients underwent electrical stimulation and EMG at the beginning and three weeks after the onset of facial palsy. This study found a significant difference between subjects according to age and normal EMG response. Also, after three weeks, significant relationships were observed between amplitude reduction and electromyography and an impaired sense of taste and improvement of symptoms and severity of disease [33]. The results of this study are in contrast to our findings. These differences yielded from a lack of follow-up and re-test after three weeks in our study. On the other hand, it may be due to differences in the aspects of follow-up and evaluation of the relationship between the severity of the disorder and taste disturbances or age at the initiation of the study.

No similar study has been performed regarding the association between Interleukin-10 and intensity of involvement in neurophysiologic examinations. Therefore, in our study, the relation between serum levels of IL-10 and historical-clinical variables was examined on the one hand, and NCS results in O. Oris and O. Oculi muscles on the other hand.

In this study, the lowest NCS O. Oculi, and O. Oris muscle amplitudes were selected, and the relevance of this ratio was evaluated to the serum level of IL-10. This survey did not lead to any significant association. Due to the lack of significance of this relation in O. Oculi and O. Oris muscles, it can be deduced that serum levels of IL-10 is not interrelated to the severity of nerve damage.

Studying the relationship between serum level of IL-10 and age, sex, affected side, family history, history of underlying disease, symptoms, abnormal taste, hyperacusis, and tearing did not show any relation between the intensity of neural injury and mentioned variables.

The relation of each prognostic group with serum IL-10 levels was assessed separately. The variables mentioned above did not have a significant relationship with each other. Considering that serum IL-10 level was not high in all patients, just in a few, low levels of IL-10 in some patients may be the consequence of dissociation of these two variables. In other words, it will be expectable that if only patients with increased serum levels of IL-10 were enrolled, there would be relations between serum level of IL-10 and neural involvement severity based on the NCS results. The most important limitation of the current study was the infeasibility of paraclinical re-evaluation and follow-up of patients for a long time. Thus, to achieve a prognostic factor, neither the clinical outcome of Bell’s palsy nor the neurophysiological parameters in the remote period were assessed in the study. Our results show only a single cross-section of IL-10 and NCS findings and assess the relationship between these measurements in the acute period of the disease.

It was already well established that IL6, IL8 and TNF-alpha levels were significantly higher in Bell’s palsy, but serum levels of these cytokines do not help to determine the prognosis in Bell’s palsy. The role of Interleukin 10 (IL10) in Bell’s palsy is yet unknown.

This cross-sectional study demonstrates that no meaningful relation exists between IL-10 serum levels and neural damage severity (based on NCS) in Bell’s palsy. Thus, serum levels of IL-10 could not be recommended as a tool for choosing a therapeutic approach, and it is not appropriate for early diagnostic confirmation and differential diagnosis of Bell’s palsy.

## Recommendations

The following suggestions are offered for the design of future studies: Measurement of serum IL-10 and NCS at the onset of the disease and during a subsequent 3-4 weeks period. A comparative study between patients and controls. Study of the relation between high serum IL-10 levels and nerve conduction abnormalities. Monitoring the concentration of IL-10 in tear and saliva at 1,3,7,14 and 21 days after involvement. Taking narrower age intervals because of different immune responses and etiology in younger patients. Measuring IL-10 at constant intervals due to immunologic cascades of cytokines being distinct based on th0e trigger and cause of the paralysis. Survey of other immunological factors concerning the clinical outcomes.

## Conclusion

There was no relationship between serum IL-10 concentration and neural involvement severity in Bell’s palsy. Therefore, the serum level of IL-10 cannot surrogate the NCS test for evaluating the severity of acute Bell’s palsy. 
